# Heat Shock Response Associated with Hepatocarcinogenesis in a Murine Model of Hereditary Tyrosinemia Type I

**DOI:** 10.3390/cancers6020998

**Published:** 2014-04-23

**Authors:** Francesca Angileri, Geneviève Morrow, Vincent Roy, Diana Orejuela, Robert M. Tanguay

**Affiliations:** Laboratory of Cell and Developmental Genetics, Department of Molecular Biology, Medical Biochemistry and Pathology, Institut de Biologie Intégrative et des Systèmes (IBIS) and PROTEO, 1030 avenue de la médecine, Université Laval, Québec G1V 0A6, Canada; E-Mails: francesca.angileri.1@ulaval.ca (F.A.); genevieve.morrow@fmed.ulaval.ca (G.M.); vincent.roy.15@ulaval.ca (V.R.); diana.orejuela@efort.org (D.O.)

**Keywords:** Hereditary Tyrosinemia type 1 (HT1), fumarylacetoacetate hydrolase (FAH), NTBC (2-[2-nitro-4-(trifluoromethyl)benzoyl]cyclohexane-1,3-dione), hepatocellular carcinoma (HCC), heat shock proteins (HSPs), apoptosis

## Abstract

Hereditary Tyrosinemia type 1 (HT1) is a metabolic liver disease caused by genetic defects of fumarylacetoacetate hydrolase (FAH), an enzyme necessary to complete the breakdown of tyrosine. The severe hepatic dysfunction caused by the lack of this enzyme is prevented by the therapeutic use of NTBC (2-[2-nitro-4-(trifluoromethyl)benzoyl]cyclohexane-1,3-dione). However despite the treatment, chronic hepatopathy and development of hepatocellular carcinoma (HCC) are still observed in some HT1 patients. Growing evidence show the important role of heat shock proteins (HSPs) in many cellular processes and their involvement in pathological diseases including cancer. Their survival-promoting effect by modulation of the apoptotic machinery is often correlated with poor prognosis and resistance to therapy in a number of cancers. Here, we sought to gain insight into the pathophysiological mechanisms associated with liver dysfunction and tumor development in a murine model of HT1. Differential gene expression patterns in livers of mice under HT1 stress, induced by drug retrieval, have shown deregulation of stress and cell death resistance genes. Among them, genes coding for HSPB and HSPA members, and for anti-apoptotic BCL-2 related mitochondrial proteins were associated with the hepatocarcinogenetic process. Our data highlight the variation of stress pathways related to HT1 hepatocarcinogenesis suggesting the role of HSPs in rendering tyrosinemia-affected liver susceptible to the development of HCC.

## 1. Introduction

Molecular chaperones play a key role as regulators of the apoptotic process [1]. Clinical data have demonstrated a correlation between increased HSPs expression and invasive potential of tumors [2,3]. Cancer cells are morphologically and functionally heterogeneous, their distinctive features make them able to survive despite an extreme environmental stress such as hypoxia, acidosis and elevated concentration of toxic metabolites. These stresses tend to generate free radicals that can damage cellular proteins. Since molecular chaperones have a protective role toward damaged proteins, it is not surprising to find them to be highly expressed in tumor cells. Most HSPs can behave as molecular chaperones for other cellular proteins participating in many cellular events with strong cytoprotective effects. HSPs have been classified into five families according to their molecular size: HSPH (HSP110), HSPC (HSP90), HSPA (HSP70), HSPD1 (HSP60), DNAJ (HSP40) and HSPB (small HSPs) [4]. Some HSPs are expressed under normal conditions (referred to as “constitutive” or “cognate”) while others are induced during stress. Among molecular chaperones, HSPA and HSPB members are strongly induced by different stresses such as heat, irradiation, oxidative stress and anticancer therapy [5]. During carcinogenesis, the transformed cells begin to express an elevated level of HSPs and this induction continues during tumor progression [2,3]. At this time, it is still unclear if the over-expression of HSPs in cancer plays a role only in supporting malignancy or if it is essential in developing the transformed phenotype [1]. In the present study, we investigated the different expression patterns of HSPs and other anti-apoptotic proteins in liver cell transformation during hepatocarcinogenesis in a murine model of hereditary tyrosinemia type 1 (HT1).

**Figure 1:**
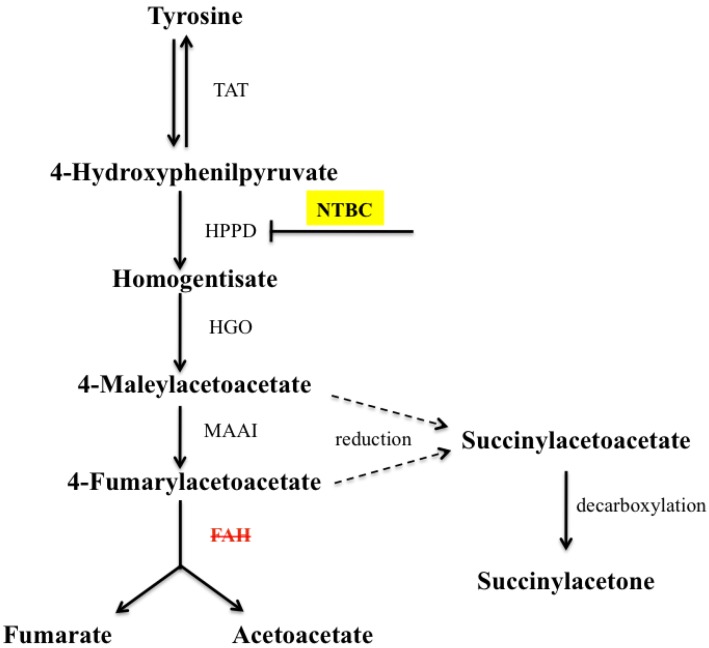
The five enzymatic steps of tyrosine catabolism and NTBC site of action. NTBC acts as a pharmacological inhibitor of 4-hydroxyphenylpyruvate dioxygenase (HPPD) and is used in therapy to avoid production of homogentisate. TAT, tyrosine aminotransferase; HGO, homogentisate dioxygenase; MAAI, maleylacetoacetate isomerase; FAH, fumarylacetocetate hydrolase.

The liver plays a central role in the pathophysiology of many inborn errors of metabolism being the main site for catabolic, synthetic and detoxification reactions. Impaired degradation of the amino acid tyrosine is a feature of several genetic diseases mainly affecting the liver. Among them HT1 (OMIM 276700) presents the most severe symptoms. HT1 is an autosomal recessive disorder characterized by severe liver damage, impaired coagulation, neurological crises, renal tubular dysfunction and a high risk of hepatocellular carcinoma [6,7,8,9,10,11]. The primary enzymatic defect in HT1 is a deficiency in fumarylacetoacetate hydrolase (FAH) activity, the last enzyme of tyrosine catabolism [12,13,14,15,16,17]. The lack of FAH produces an accumulation of the toxic upstream metabolites fumarylacetoacetate (FAA), maleylacetoacetate (MAA), and succinylacetone (SA) (Figure 1). This condition is responsible for the progressive injury to hepatocytes leading to chromosomal instability, and cell death in animals, and cultured cell models of the disease [18,19,20,21,22,23]. The only available treatment for this disease is the combination of a diet low in tyrosine and phenylalanine and a daily intake of NTBC (2-[2-nitro-4-(trifluoromethyl)benzoyl]cyclohexane-1,3-dione) also known as nitisinone (Orfadin^®^, Swedish Orphan Biovitrum, Stockholm, Sweden). This drug inhibits 4-hydroxyphenylpyruvate dioxygenase (HPPD), one of the enzymes involved in the catabolic pathway upstream of FAH, and therefore prevents the formation of toxic products responsible for liver damage (Figure 1). The efficacy of this therapy has improved the liver disease associated with FAH deficiency. However clinical data indicate that NTBC provides only partial protection against liver dysfunction. Indeed despite the improvement in survival and quality of life with NTBC treatment, HT1 remains a chronic disorder with several long-term complications, a persistent even though lower risk of HCC, and possible subnormal intellectual functions [24,25,26,27].

In mice HT1 is lethal but can be rescued by blocking upstream steps in the tyrosine catabolic pathway (*i.e*., NTBC). It has been reported that a chronic HT1 stress, induced by withdrawal of NTBC, results in resistance to cell death *in vivo* as shown by the survival of *fah^−/−^* mice to an otherwise lethal dose of homogentisate [28]. This resistance led us to investigate how the balance between cell death and survival signals is maintained or disrupted in a murine FAH-deficient model of HT1. Therefore, we analyzed the relationship between the evolution of major survival and apoptotic pathways and the progression of clinicopathologic features of the disease. Our previous studies showed that the HT1 phenotype induced by NTBC-withdrawal causes a cellular insult eliciting the ER stress response and increasing the level of stress-related proteins [29]. This hepatic stress causes the activation of many survival pathways and inhibits the intrinsic apoptotic cascade promoting hepatocarcinogenesis [30]. Thus, to clarify the biologic pathways that degenerate the HT1 stress response to cancer, we examined the gene expression patterns in tumoral liver fractions compared to healthy livers. Our studies show how the survival state in HT1 livers under progression of cancer promoted by long-term NTBC withdrawal, can influence the expression of HSPs and other pro-survival proteins that act at the mitochondrion level. Indeed, molecular chaperones have multiple roles in cell survival, that depend on their distinctive features, one of them being their interactions with anti-apoptotic proteins of the BAG and BCL-2 families [31].

## 2. Results and Discussion

### 2.1. Gene Expression Analysis in fah^−/−^ Mice after NTBC Withdrawal

**Figure 2:**
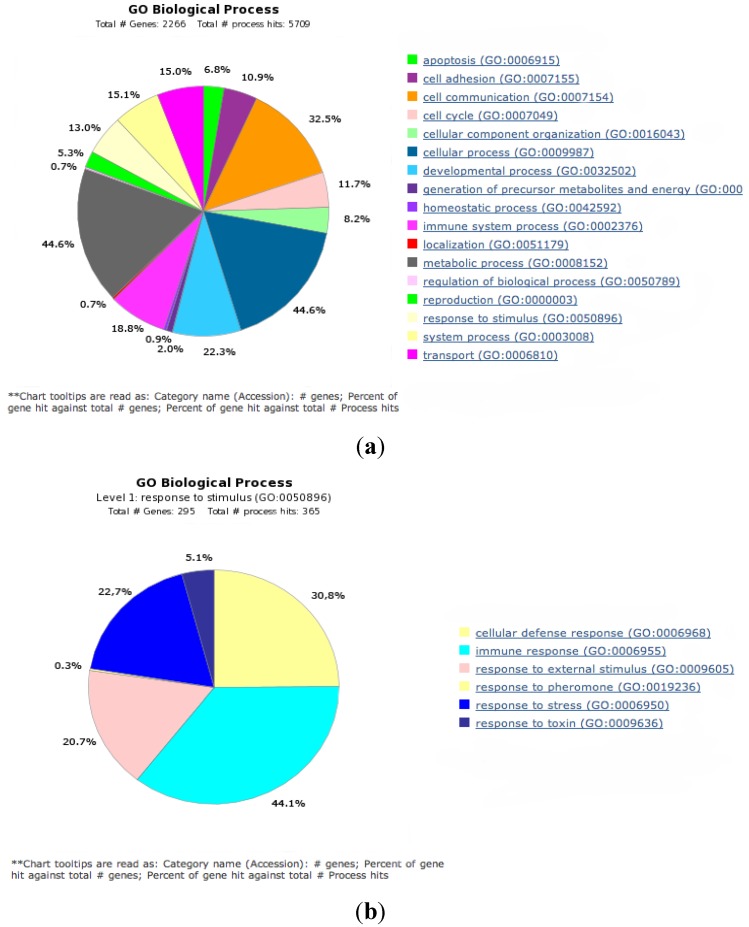
(**A**) Biological processes regulated by all significant differentially expressed genes assessed by Gene Ontology (GO) search and summarized according to their functions (PANTHER classification system); (**B**) The GO level 1 of the response to stimulus was further mined down showing as subset the percentage of molecules involved in stress response.

**Figure 3:**
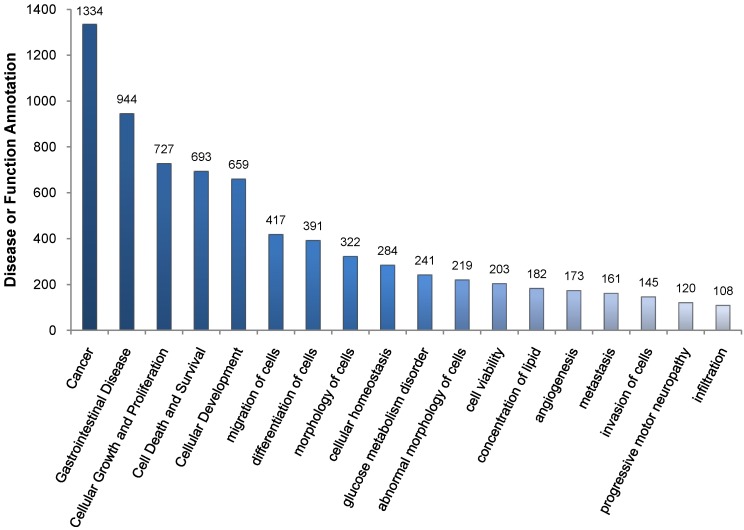
Disease and function annotation according to IPA software. As it can be noticed the largest amount of deregulated genes is involved in the mechanism of cancer. Others relevant processes are those implicated in gastrointestinal disease, cellular growth and proliferation, migration and differentiation of cells. These evidence suggest that under HT1 stress there is a wide gene regulation involving many biological processes that all together might foster the tumoral progression.

The murine FAH-deficient model of HT1 presents a neonatal lethal phenotype that can be rescued by treating the pregnant and nursing females and the pups with NTBC [32]. FAH deficient mice have a severe phenotype, displaying rapidly a remarkable impairment of vital functions upon withdrawal of the drug. The *fah^−/−^* mice receive NTBC in drinking water at 7.5 mg/L from birth. Assuming that they drink 3–5 mL of water per day, the mice are kept on a high-dose treatment. Our previous studies demonstrate the changes in the activation state of cellular signaling pathways in response to HT1 stress that have been observed after one to five weeks of NTBC withdrawal [30]. With the aim to assess the different patterns of genes expression during a long-term HT1 stress characterized by tumor development, we performed differential gene expression profile in livers of tyrosinemic mice having developed HCC following cumulative HT1 stress after a period of NTBC interruption from 5 to 7 weeks relative to livers of wild type and *fah^−/−^* control mice (Figure 2). Selective dissection of tumor and non-tumor tissues and extraction of total RNA allowed the analysis of gene expression with DNA microarrays of Whole Mouse Genome (Agilent Technologies, Santa Clara, CA, USA). We investigated the modulation of genes and their change in tumoral fractions compared with *fah^+/+^* and NTBC-treated *fah^−/−^* mice. Differently expressed genes were selected by filtering with confidence at *p* ≤ 0.05, considering those with a difference in level of expression of at least 2-fold. Biological classification of differently regulated genes was obtained by Gene Ontology (GO) analysis (PANTHER classification system). Tumoral samples displayed significant modulation in the expression of genes leading to cell survival. The GeneSpring algorithm (Agilent) and the IPA software (Ingenuity, QIAGEN, Redwood City, CA, USA) were further used to create functional classes of genes implicated in regulation of the survival pathway in the context of HT1 (Table S1, Supplementary Data). The wide range of genes deregulated in the tumoral fraction included a substantial number of genes implicated in cellular growth and proliferation, organismal survival and apoptosis, differentiation, inflammation and cell migration (Figure 3 and Table S1, Supplementary Data). These results show that in tumor cells a preferential expression of specific constellation of genes related to hepatocarcinogenesis occurs.

### 2.2. HSPs Expression Is Associated to HT1 Progression in Mice

**Figure 4:**
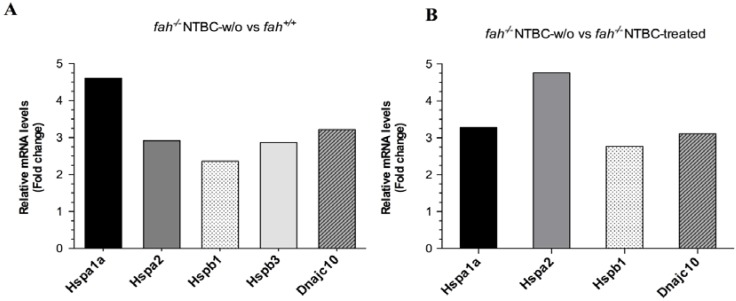
HSPs genetic expression. Influence of HT1 stress on expression levels of transcripts codifying for HSP70 family (*Hspa1a*, *Hspa2*), HSPB family (*Hspb1*, *Hspb3*) and DNAJ co-chaperone family. Graphics showing the relative expression level in livers after withdrawal of NTBC in *fah^−/−^*mice compared to livers of wild type (**A**), and of *fah^−/−^* NTBC-treated mice (**B**). Data are given as mean of two experiments (*n* = 2) in a single scan. Each sample is representative of a pool of four different mice for each condition. For all up-regulated genes *p* < 0.05 was obtained by variance analysis (ANOVA). The tumoral fractions showed an up regulation in the HSPs families up to 5-fold higher compared to the *fah^+/+^* (**A**) and *fah^−/−^* control (**B**) mice.

Notably, among genes deregulated in tumoral fractions we found those belonging to HSP families. Liver samples under HT1 stress showed over-expression of HSPs transcripts; among them, *Hspb1* and the HSP70 member *Hspa1a* and *Hspa2* were the most up-regulated compared to wild type (Figure 4A) and control mice (Figure 4B). Following NTBC withdrawal, *Hspa1a* showed a 4.61 fold increase in *fah*^−/−^ NTBC-w/o mice compared to *fah^+/+^* mice and 3.28 fold compared to *fah^−/−^* NTBC-treated mice. *Hspb1* was also higher in mice after NTBC withdrawal presenting fold changes of 2.36 and 2.77 compared to *fah^+/+^* and NTBC-treated *fah^−/−^* respectively (Figure 4). Other transcripts of the HSP family were also induced such as *Hspa2*, *Hspb3* and *Dnajc10*. *Hspa2* encodes heat shock-related 70 kDa protein 2 involved in normal development of germ cell and is over expressed in cancer cells [33,34], bladder urothelial carcinoma [35] and peptic ulcer [36].

**Figure 5:**
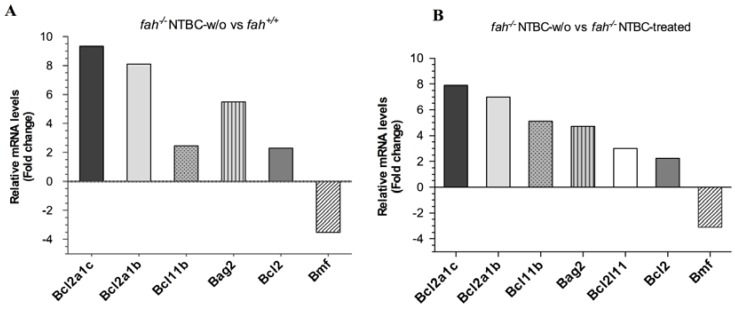
BCL-2 related protein mRNAs expression in tumoral liver tissue. Comparison of gene expression values of the BCL-2 family members between healthy and tumoral livers. Graphics showing the relative expression level in livers after withdrawal of NTBC in *fah^−/−^* mice compared to liver of wild type *fah^+/+^* mice (**A**), and *fah^−/−^* NTBC-treated mice (**B**). Data are given as mean of two experiments (*n* = 2) in a single scan. Each sample is representative of a pool of four different mice for each condition. For all deregulated genes *p* < 0.05 was obtained by variance analysis (ANOVA). A ratio up to 9-fold higher of the BCL-2 related protein mRNAs is observed. In particular the anti-apoptotic *Bcl2a1a*, *Bcl2a1b*, *Bcl2*, *Bcl2l11*, were over-expressed, while the pro-apoptotic *Bmf* was down-regulated.

The *Hspb3* gene encodes for HSPB3 (HSPL27), a small heat shock protein involved in distal motor neuropathy [37]. Unlike the other HSPs members, *Hspb3* was significantly deregulated in *fah^−/−^* tyrosinemic mice compared to *fah^+/+^* wild type mice, but no difference was observed when compared to *fah^−/−^* NTBC-treated mice (Figure 4). *Dnajc10* is a member of the HSP40 family and encodes for the endoplasmic reticulum resident protein DNACJ10 shown to be up-regulated after ER-stress [38,39]. Since the functions of HSPs in tumorigenesis have been related to their ability to interfere with the apoptotic machinery, we also examined the level of mRNAs encoding anti-apoptotic proteins that act at the mitochondrial level. A subset of transcripts of the BCL-2 family was found to be over-expressed in tumors of HT1 mice (Figure 5). In particular the anti-apoptotic members of the BCL-2 like family, *Bcl2a1a*, *Bcl2a1b*, *Bcl2l11 and Bcl2*, showed a remarkable fold change compared to *fah^+/+^* and *fah^−/−^* NTBC-treated (Figure 5). Moreover, *Bag2* gene, member of the BCL-2 athanogene related proteins, showed an expression value respectively of 5.49 fold and 4.73 fold higher in wild type (A) and NTBC-treated mice (B)*.*

### 2.3. Increase of Pathological Feature during Long-Term HT1 Stress Promotes HCC Development

**Figure 6:**
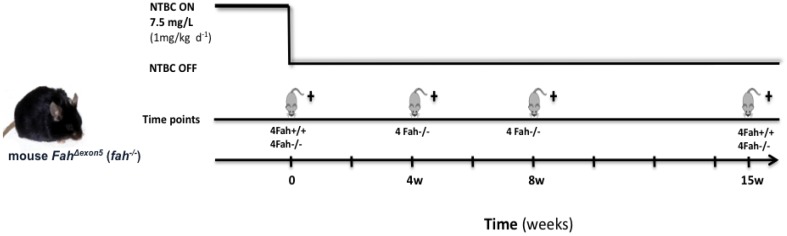
Protocol of study.

In the case of the NTBC withdrawal induced HT1 stress, the physiological state of the *fah^−/−^* mouse reflects a constant disease progression, proportional to the damage accumulation. Several studies on the role of HSPs in tumorigenesis have established that inducible HSPs are up-regulated in cancer cells. HSPs have also been involved in facilitating cell survival through the inhibition of apoptosis [40], and cell senescence [41]. Hence the result of gene expression analysis prompted us to investigate the participation of molecular chaperones in the biological processes that degenerate the HT1 stress into cancer. Thus, we built a new protocol with the purpose of inducing a progressive severe phenotype of the disease in which 4-months old *fah^−/−^* mice were withdrawn from NTBC treatment for periods up to 15 weeks. Figure 6 shows the time points of the protocol chosen to evaluate the different grades of injury post-withdrawal.

**Figure 7:**
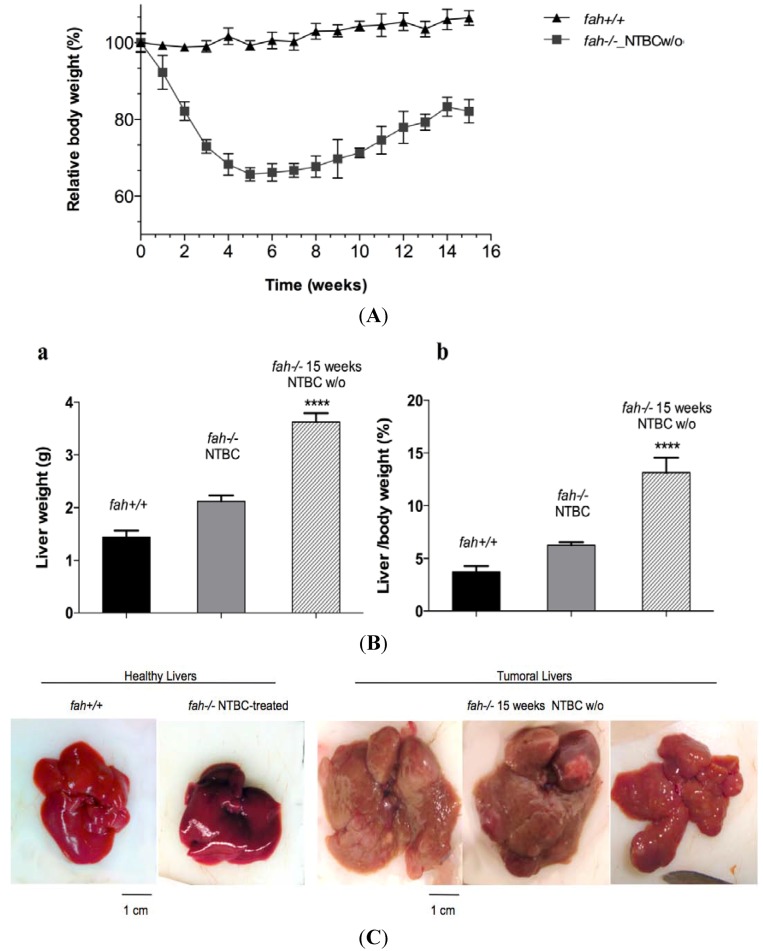
Changes in body mass values (%) of wt (▲) and *fah^−/−^* (■) mice following NTBC withdrawal. (A) Body weight curves of *fah^+/+^* (▲), and *fah^−/−^* NTBC w/o (■) mice. Each time point indicates the average of four mice, with error bars indicating standard deviation. Unpaired t-test (Welch’s correction) was applied for statistical validation of the two groups (*p* value < 0.0001 for all the time point). (**B**) Liver weight (**a**), and liver /body weight ratios (**b**), of *fah^+/+^*, *fah^−/−^* NTBC-treated mice and *fah^−/−^* mice following 15 weeks of withdrawal (*n* = 4, **** *p* < 0.0001, ANOVA variance analysis). (**C**) Representative pictures of whole healthy livers and tumoral livers. The images were taken at the beginning of the procedure (control mice) and after 15 weeks of withdrawal from treatment.

Four time points were set to look at the disease in different stages until formation of large tumors in 100% of the mice at 15 weeks. Young *fah^−/−^* mice were treated from birth until the age of four months with NTBC. As control, *fah*^+/+^ healthy mice were used. After four months of NTBC treatment the drug was removed to allow HT1 development and we started the harvest. The mice were examined daily and any variation of physiological conditions was recorded. The animals were weighed three times a week until the end of the protocol (15 weeks). As previously reported, the *fah* knockout mice displayed a severe weight loss and a rapid hepatic dysfunction following the drug withdrawal. After five weeks without NTBC, *fah^−/−^* mice display a loss of total body mass reaching ~38% from initial mass (Figure 7A). During the next 10 weeks they regain weigh up to ~80% of the original body mass until the time of sacrifice. This gain of weight was mainly due to the liver that doubled its mass and presented many macronodules (Figure 7B,C**)**. The resulting slope of total weigh was a sign of clinical illness leading to poor prognosis. Indeed, all mice harvested at the end of the protocol had the tendency to develop a severe tumor phenotype (Figure 7C). Histological analysis of the livers at the different time points showed a progressive hepatic pathology with inflammation, severe histological lesions, architectural rearrangements, severe hepatocellular changes, apoptosis, and many erythropoietic foci (data not shown).

### 2.4. Variation in the Expression of HSPA1A and HSPB1 Is Correlated to HT1 Progression in NTBC-withdrawn Mice

**Figure 8:**
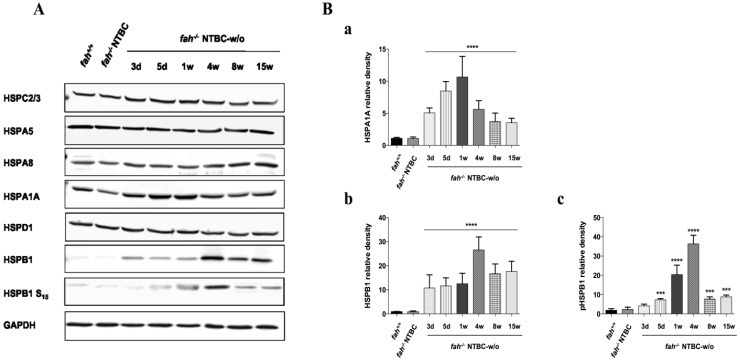
Western blot analysis of heat shock proteins. (**A**) Variation in the levels of anti-apoptotic HSPB1 and HSPA1A, in liver of *fah^+/+^*, *fah^−/−^* NTBC-treated and *fah^−/−^* NTBC-off mice. HSPC2/3, HSPA5, HSPA8 and HSPD1 were also tested as HSP family members. Data are representative of at least two different experiments with four biological replicates; (**B**) Histograms show quantitative representation of the level of HSPs proteins after normalization to levels of GAPDH and the fold-change protein level expression is reported in comparison to *fah^+/+^* mice. Data are mean ± standard deviation for four samples. Significance of group differences was evaluated using ANOVA for multiple comparisons (*** *p* < 0.001 and **** *p* < 0.0001). Where statistical significance was not observed after densitometric analysis, graphics were not produced (*i.e*., HSPC2/3, HSPA5, HSPA8, HSPD1).

Liver homogenates obtained from mice taken off NTBC from one to 15 weeks, were next used to determine the level of stress induction and resistance to apoptosis. HSPC2/3 (HSP90 α/β), HSPD1 (HSP60) and HSPA5 (BiP) showed a constant level of expression during disease progression (Figure 8A). The cognate form of the 70kD heat shock protein, HSPA8, remains almost unvaried in *fah^−/−^* mice, independently of the NTBC treatment, with a faint reinforcement in the advanced hepatocarcinogenetic stage. In contrast, the inducible HSP70 member HSPA1A and the small HSPB1 were significantly deregulated along the entire experience (Figure 8A,B).

HSPA1A shows a remarkable variation in its expression during the starvation from the drug. It is already expressed in *fah^+/+^* and *fah^−/−^* NTBC-treated mice. Soon after the withdrawal it goes up for the first week, then it starts to come back to the original level (Figure 8A,B(a)). HSPB1 and HSPA1A proteins have an anti-apoptotic role in cells by inhibiting the formation of the apoptosome, thus eliciting the intrinsic apoptotic pathway. Induction of HSPB1 appears already three days after NTBC-treatment interruption and increases notably during HT1 stress progression with a reinforcement at four weeks of withdrawal. Its phosphorylated form on serine 15 appears also increased in the same proportion (Figure 8A,B(b,c)) which is different from what has been reported in some experimental studies where an attenuated phosphorylation of HSPB1 in advanced HCC was observed [42,43]. The phosphorylated form of HSPB1 at serine 15 is involved in the ability of this chaperone to switch between small and large oligomers in order to accomplish its anti-apoptotic functions [40,44,45,46,47]. As reported by other authors the equilibrium between large and small oligomers might be shifted by phosphorylation, depending on the physiological requirements of the cell. Interestingly, the ability to form small oligomers upon phosphorylation was shown to mediate the entry of HspB1 in the nucleus [48,49,50,51]. Based on these findings HSPB1 phospho-Ser-15 might perform other important functions in HT1-dependent tumorigenesis by entering into the nucleus as small oligomer. Further studies will be needed to investigate the crosstalk between aggregated and small-dissociated forms that could regulate HSPB1 sub-cellular localization and biological functions in liver cancer during HT1 stress. In this murine model the chaperone-induced cytoprotection could rescue cells from apoptosis, easing the deleterious consequences of HT1 chronic stress. It is also important to notice the variability of expression in the HSPs family in the different stages of the disease. In fact, as previously reported, HSP70 and HSPB1 could be involved in different phases of carcinogenesis implicating a complex network of apoptotic pathways, supporting the survival state and favoring the progression of the malignancy. Tumoral cells could use the cytoprotective effect of HSP70 and HSPB1 and their ability to interact with many targets, to survive to the HT1-driven environmental stress promoting the tumoral invasion.

### 2.5. Survival State in the Liver Is Reinforced by Increased Expression of the Anti-Apoptotic BCL-2 Protein

**Figure 9:**
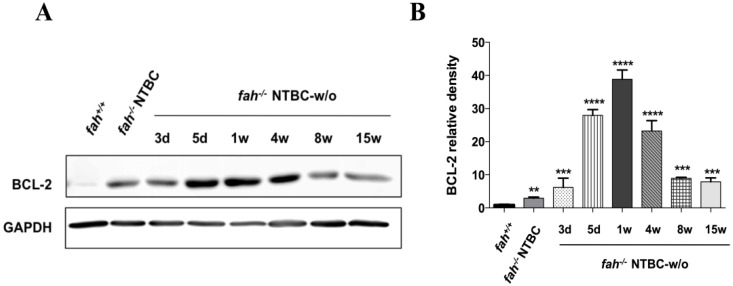
BCL-2 protein expression in HT1. (**A**) Levels of BCL-2 in livers of *fah^+/+^*, *fah^−/−^* NTBC-treated and *fah^−/−^* NTBC-off mice; (**B**) Histogram shows quantitative representation of the BCL-2 protein level after normalization to levels of GAPDH. Fold-change protein level expression is reported in comparison to *fah^+/+^* mice. Data are mean ± standard deviation for four samples. Significance of group differences was evaluated using ANOVA for multiple comparisons (** *p* < 0.01, *** *p* < 0.001, **** *p* < 0.0001).

In agreement with the microarray result and with our previous studies [30], the levels of the anti-apoptotic BCL-2 protein were also affected (Figure 9). BCL-2 is the central coordinator of apoptotic events into the mitochondria. The rupture of the outer mitochondrial membrane induces the mitochondrial translocations and multimerization of the pro-apoptotic BCL-2-associated X protein (BAX) resulting in the leakage of cytochrome c and other mitochondrial proteins for the execution phase of apoptosis. In this context BCL-2 plays a fundamental role by preventing the mitochondrial generation of ROS and dimerizing with BAX, thus blocking the BAX interaction with the voltage-dependent anion channel (VDAC) to form pores that are large enough to allow the passage of cytochrome *c* [52,53]. In the HT1 murine model the protein levels of the anti-apoptotic BCL-2 show a remarkable variation with the progression of the disease (Figure 9). Indeed, BCL-2 was undetectable in *fah^+/+^* mice, but it is already expressed in the *fah^−/−^* control, increasing its expression in the first week up to four weeks without treatment, and then starting to decrease its level again (Figure 9A,B). The trend of BCL-2 seems to be comparable to HSPA1A as it tends to increase with the progression of the disease but, in contrast to HSPA1A, it seems to continue until the fourth week of withdrawal and then decrease to the beginning level. Over expression of BCL-2 has already been found in various types of cancer [54] and it is considered as a biomarker of resistance to chemotherapy and radiotherapy [55,56,57] although its universal role in cancer is still unclear [58]. These results suggest the involvement of BCL-2 together with the HSPs, in the biological series of events that exacerbate the HT1 stress leading to hepatocarcinogenesis. Herein we could conclude that in the first phase of HT1 stress BCL-2 contributes, cooperating with HSPs, in the apoptosis resistance that facilitates tumor onset.

### 2.6. HSPs and Tumor: An Intricate Co-Operation

From these results we suggest that HT1 related liver cancer could arise from a complex network of gene deregulation due to the metabolic defect. Activation of many survival pathways has already been reported in HT1 cells models [20], and resistance to cell death was also documented in HT1 stress [28,29]. In these studies an induction of HSPB1 and HSPA1A protein levels has been observed in *fah^−/−^* mice at different periods after NTBC withdrawal. It has been shown that HSPs can participate in multiple cellular processes pertinent to tumor aggressiveness promoting cancer development by increasing cellular migration [59] and differentiation [60]. Although these HSPs are not mediators of proliferation, their role in tumorigenesis involves their ability to block programmed cell death (PCD). They have been shown to play an essential role in the survival of a wide number of human cancer cells by interfering with multiple apoptotic pathways. HSPA1A and HSPB1 have a strong cytoprotective effect that increases metastatic potential and growth ability in tumor cells, as a consequence of the inhibition of the apoptotic machinery [61,62]. These HSPs can regulate in an independent manner the apoptotic cascade upstream of the mitochondria by inhibiting stress inducible signals, at mitochondrial level by preventing membrane permeabilization and release of cytochrome *c,* and by suppressing caspase activation downstream the mitochondria [63]. HSP70 has also been implicated in other mechanisms of cell death in addition to PCD. This HSP has been shown to increase the lysosomal resistance against chemical and physical membrane destabilization [64]. Nevertheless it has been suggested that HSP70 could modulate JNK and ERK phosphorylation in apoptosis induced by hyperosmolarity [65] and promote the stabilization of phosphorylated form of PKC [66].

HSPB1 is an ATP-independent chaperone and interacts with its substrates by forming multimeric complexes [67]. The oligomerization of HSPB1 is a dynamic process highly regulated by phosphorylation at different serine residues (Ser15, Ser78, Ser82 in humans; Ser15 and Ser86 in mice) and involved in the determination of substrate affinity. Experimental evidence suggest that HSPB1 mainly acts as an inhibitor of caspase-dependent apoptosis and this action seems to be related with large unphosphorylated oligomers [45,47]. However the direct interaction with cytochrome *c* does not require the phosphorylation of HSPB1 [44]. Still, small-phosphorylated oligomers of HSPB1 efficiently bind Daxx and F-actin enhancing its cytoprotective functions [40,46].

Consistent with all these observations, these inducible chaperones have been implicated in cancer progression and in chemotherapy resistance and their overexpression is considered as negative prognostic marker for different cancers, as colorectal cancer [68,69,70], intrahepatic cholangiocarcinoma [71], prostate cancer [72] and urothelial carcinoma [73]. Furthermore, with the aim to find new strategies to block tumoral invasion, HSPB1 and HSP70 have been proposed as target for anticancer therapy [74,75]. Our results show that transcripts encoding specific HSPs and the corresponding proteins are induced in HT1 livers during carcinogenesis. This might lead to further investigations to understand the role that these HSPs may have as prognostic biomarkers for HCC development in HT1 patients and in developing new therapies in addition to NTBC treatment in these patients.

HSPs and BCL-2 families regulate cell survival through many interactions that depends on their unique features. Tumor cells may escape from caspase-mediated apoptosis by overexpressing anti-apoptotic proteins. Thus our investigations were also extended to the level of proteins that control the cell’s response to apoptosis. Regulation of the mitochondrial pathway of apoptosis via BCL-2 family members is supported by gene and protein expression. BCL-2 family consists of about 25 members and includes pro-apoptotic factors such as BAD, BAK, BAX, BID, BIM and anti-apoptotic proteins including BCL-2 and BCL-X_L_. The balance between members of BCL-2 family governs cell’s response to apoptosis signaling. Altered expression of the survival genes such as those codifying for BCL-2 anti-apoptotic proteins induces dysfunction in the apoptotic machinery and has therefore been associated with carcinogenesis [76,77,78,79,80,81,82]. Among genes up-regulated in HT1 tumoral fractions we noticed also one transcript of the BAG family member, BAG-2 (Figure 5). The BAG family (BCL-2 associated athanogene) represents nucleotide exchange factors in the ATP-dependent chaperone cycle of HSP70 [83,84,85]. They make a direct link between HSP and BCL-2 helping BCL-2 activation [86]. BAG family members possess an ubiquitin-like domain that allows their association with the proteasome [87,88]; this cooperation not only stimulates chaperone-assisted degradation but also interferes with it. In particular, BAG2 was reported to inhibit the CHIP-mediated ubiquitylation of HSP70, despite other BAG co-chaperone members, such as BAG-1, that promote the release of HSP70 in the vicinity of the proteasome stimulating the HSP70 degradation [89]. We need to investigate more about this involvement in HT1 stress but it is nevertheless tempting to speculate that the cooperation between HSPs, BCL-2 and BAG family members exerts regulatory function contributing to the cell death resistance that promote carcinogenesis in HT1 stressed cells.

## 3. Experimental

### 3.1. Animal Maintenance and Treatments

HT1 phenotype was reproduced using homozygous FAH deficient mice (*fah^∆^*^exon5^, referred as *fah^−/−^*) [27]. All animals were originally inbred of 50% 129SvJ and 50% C57BL/6 strains. The *fah* knockout mice and normal mice *fah^+/+^* were maintained in a temperature and humidity-controlled environment with a 12 h light-dark cycle. Mice were allowed access to rodent food (0.82% tyrosine, 0.51% phenylalanine, Purina 5075-U.S., Charles River, St-Constant, QC, Canada) and drinking water, which was supplemented with 2-(2-nitro-4-trifluoromethylbenzoyl)-1,3-cyclohexanedione, NTBC (kindly provided by S. Lindstedt, Gottenburg University, Sweden) for the *fah^−/−^* mice. The NTBC was used at a concentration of 7.5 mg/L and provided from fetal stage by treatment of the pregnant and nursing females. To induce the HT1 phenotype mice were withdrawn from NTBC treatment for different periods of time from 1 to 15 weeks. Mice control *fah^+/+^* and mice *fah^−/−^* on NTBC were used to underline the differences dues to genotypes. Mice were treated according to the guidelines of the Canadian Council of Animal Care (CCAC) and euthanized at the time of sacrifice by cardiac puncture with an overdose of ketamine/xylazine (0.2 mL/10g).

### 3.2. Gene Expression Analysis

Total RNA was isolated from snap-frozen liver tissues using TRIzol Plus Reagent (Invitrogen Corporation, Burlington, ON, Canada) according to the manufacturer’s instructions. Total RNA was treated with DNase using the RNase-free DNase kit and RNeasy spin column (QIAGEN, Toronto, ON, Canada) and dissolved in RNase-free water to a final concentration 0.2–0.5 μg/μL. The quality and purity of RNA samples was checked by capillary electrophoresis using the Agilent 2100 Bioanalyzer and Agilent RNA Nano 6000 LabChip kits (Agilent Technologies). Three different RNA pools resulting from four different mice livers were used to perform the hybridization on the four arrays. Five hundred nanograms of purified RNA were used to prepare cDNA labeled with Cy3 and Cy5, using the two colors Agilent Low Input Quick Amp Labeling kit, and hybridized using Agilent Two-Color Microarray-based gene expression platform using the 4x44K Two Colors Mouse Whole Genome Microarray, 41,534 genes (Agilent Technologies). Two hybridizations were carried out for tumor against reference samples using a fluorescent dye reversal technique (dye-swap). Slides were scanned using an Agilent DNA Microarray Scanner and Cy3/Cy5 intensity data were extracted using Agilent’s Feature Extraction software (Agilent Technologies). Transcriptome analysis was performed using Agilent Genespring GX11 software (FRSQ-Réseau Cancer Core Genome Facility, Hôtel-Dieu, Quebec, QC, Canada). A list of differently expressed genes was first created, by filtering on confidence at *p* ≤ 0.05, consecutive lists were generated considering a fold change value more then 2-fold. IPA Ingenuity software (QIAGEN, Redwood City, CA, USA), was further used to create functional classes of genes implicated in regulation of the survival pathway in the context of HT1. Biological processes regulated by all significant differentially expressed genes were classified according to their function using the Gene Ontology (PANTHER^™^ GO slim).

### 3.3. Protein Electrophoresis and Western Blot Analysis

Liver tissues were immediately snap frozen in liquid nitrogen and stored at −80 °C. For western blot, frozen tissues were homogenized in 1× RIPA Buffer (20 mM Tris-HCl, pH 7.5; 150 mM NaCl; 1 mM Na_2_EDTA; 1 mM EGTA; 1% NP-40) with 1X protease inhibitor cocktail tablets (cOmplete, Mini, EDTA-free; Roche Diagnostics, Indianapolis, USA) and 1× phosphatase inhibitor cocktail tablets (PhosSTOP; Roche Diagnostics, Indianapolis, IN, USA). The homogenized extracts were centrifuged at 10,000 *g* for 15 min at 4 °C. Protein concentration was measured with the Bio-Rad Protein Assay (Bio-Rad Laboratories Inc., Hercules, CA, USA). Proteins were resolved by electrophoresis on a 12% SDS-PAGE gel, transferred onto a nitrocellulose blotting membrane (BioTrace NT, Pall Life Ltd., St-Laurent, QC, Canada), blocked with 5% non-fat dried milk in TBS (Tris-buffered saline; 50 mM Tris, 150 mM NaCl, pH 7.5) and probe overnight at 4 °C with primary antibodies following recommendations by the manufacturer. Home-made rabbit polyclonal antibodies against HSPA1A (#799 1:10,000), HSPA8 (#1477 1:10,000), HSPC2/3 (#214 1:10,000) and HSPD1 (#37 1:10,000), were used and are described in Tanguay *et al.* [90]. Rabbit polyclonal antibody against HSPB1 was kindly provided by J. Landry (#L2R3 1:1000, Hôtel-Dieu Research Center, CHU-Q, Quebec, QC, Canada). Rabbit polyclonal antibody against HSPB1 phospho-Ser15 and HSPA5/BiP were purchased from Stressgen Biotechnologies Corp. (Victoria, BC, Canada) (cat. #SPA-525 1:1000, and #SPA-826 1:1000). Monoclonal rabbit antibodies against BCL-2 and GAPDH (cat. #2870 1:1000 and #5174 1:2000) and secondary anti-rabbit IgG-HRP (cat. #7074 1:2000) antibody were from Cell Signaling Inc. (Beverly, MA, USA). Odyssey^®^ Infrared Imaging System (Li-COR, Biosciences, Lincoln, NE, USA) was used for proteins detection. Densitometric analysis were performed on western blot images using ImageJ 1.47v. The obtained values were normalized to GAPDH and finally were compared to the control *fah*^+/+^ mice. Data in histograms indicate the average of four mice for each time point, with error bars indicating standard deviation.

### 3.4. Statistical Analysis

GeneSpring GX11 software (Agilent Technologies) was used to generate a list of selected genes and for different statistical methods on gene expression data. The significance of group differences was evaluated using ANOVA multivariance analysis, followed by post hoc Tukey test for selection of genes in pairwise comparison. Unpaired Student’s t-test was used in comparing weight variation of *fah^+/+^* and *fah^−/−^* NTBC-withdrawn mice during the protocol of study. Ordinary one-way ANOVA variance analysis (Brown-Forsythe test) was applied to measure the statistical significance of the trend of liver weight and liver/body weight ratio in the different groups. Statistical evaluation on western blot images was performed by two-way analysis of variance (ANOVA) followed by Dunnett’s multiple comparison test (GraphPad Prism 6.0d). A statistically significant difference was defined as * *p* < 0.05, ** *p* < 0.01, *** *p* < 0.001, **** *p* < 0.0001. All *p* values were derived from two-tailed tests.

## 4. Conclusions

In summary, our results demonstrate that HT1 stress induces progressive damage on hepatic system as soon as the HT1 stress is initiated by NTBC withdrawal, concomitant to an early, constant and specific activation of the survival pathway in livers of *fah^−/−^* mice. In early and in advanced carcinogenesis, these pathways seem to be differently modulated suggesting that more detailed investigations are needed to elucidate whether they are simply fast-acting proteins in response to tumor stress or indeed, specific marker for different phases of cancer progression and development.

## Supplementary Files

Supplementary File 1 Supplementary Data (PDF, 133 KB)

## References

[1] Orejuela D., Bergeron A., Morrow G., Tanguay R.M., Calderwood S.K. (2007). Small heat shock proteins in physiological and stress-related processes. Cell Stress Proteins.

[2] Ciocca D.R., Arrigo A.P., Calderwood S.K. (2013). Heat shock proteins and heat shock factor 1 in carcinogenesis and tumor development: An update. Arch. Toxicol..

[3] Mjahed H., Girodon F., Fontenay M., Garrido C. (2012). Heat shock proteins in hematopoietic malignancies. Exp. Cell Res..

[4] Kampinga H.H., Hageman J., Vos M.J., Kubota H., Tanguay R.M., Bruford E.A., Cheetham M.E., Chen B., Hightower L.E. (2009). Guidelines for the nomenclature of the human heat shock proteins. Cell Stress Chaperones.

[5] Garrido C., Gurbuxani S., Ravagnan L., Kroemer G. (2001). Heat shock proteins: Endogenous modulators of apoptotic cell death. Biochem. Biophys. Res. Commun..

[6] Weinberg A.G., Mize C.E., Worthen H.G. (1976). The occurrence of hepatoma in the chronic form of hereditary tyrosinemia. J. Pediatr..

[7] Kim S.Z., Kupke K.G., Ierardi-Curto L., Holme E., Greter J., Tanguay R.M., Poudrier J., D’Astous M., Lettre F., Hahn S.H. (2000). Hepatocellular carcinoma despite long-term survival in chronic tyrosinaemia I. J. Inherit. Metab. Dis..

[8] Kvittingen E.A. (1991). Tyrosinaemia type I—An update. J. Inherit. Metab. Dis..

[9] Van Spronsen F.J., Thomasse Y., Smit G.P., Leonard J.V., Clayton P.T., Fidler V., Berger R., Heymans H.S. (1994). Hereditary tyrosinemia type I: A new clinical classification with difference in prognosis on dietary treatment. Hepatology.

[10] Van Spronsen F.J., Bijleveld C.M., van Maldegem B.T., Wijburg F.A. (2005). Hepatocellular carcinoma in hereditary tyrosinemia type I despite 2-(2 nitro-4–3 trifluoro- methylbenzoyl)-1, 3-cyclohexanedione treatment. J. Pediatr. Gastroenterol. Nutr..

[11] Mitchell G., Larochelle J., Lambert M., Michaud J., Grenier A., Ogier H., Gauthier M., Lacroix J., Vanasse M., Larbrisseau A. (1990). Neurologic crises in hereditary tyrosinemia. N. Engl. J. Med..

[12] Lindblad B., Lindstedt S., Steen G. (1977). On the enzymic defects in hereditary tyrosinemia. Proc. Natl. Acad. Sci. USA.

[13] Kvittingen E.A., Jellum E., Stokke O. (1981). Assay of fumarylacetoacetate fumarylhydrolase in human liver-deficient activity in a case of hereditary tyrosinemia. Clin. Chim. Acta.

[14] Tanguay R.M., Valet J.P., Lescault A., Duband J.L., Laberge C., Lettre F., Plante M. (1990). Different molecular basis for fumarylacetoacetate hydrolase deficiency in the two clinical forms of hereditary tyrosinemia (type I). Am. J. Hum. Genet..

[15] Phaneuf D., Lambert M., Laframboise R., Mitchell G., Lettre F., Tanguay R.M. (1992). Type 1 hereditary tyrosinemia. Evidence for molecular heterogeneity and identification of a causal mutation in a french canadian patient. J. Clin. Invest..

[16] Mitchell G.A., Grompe M., Lambert H., Tanguay R.M. (2001). Hypertyrosinemia. The Metabolic and Molecular Bases of Inherited Diseases.

[17] Knox W.E, Edwards S.W. (1955). Enzymes involved in conversion of tyrosine to acetoacetate. Methods Enzymol..

[18] Jorquera R., Tanguay R.M. (1997). The mutagenicity of the tyrosine metabolite, fumarylacetoacetate, is enhanced by glutathione depletion. Biochem. Biophys. Res. Commun..

[19] Jorquera R., Tanguay R.M. (1999). Cyclin b-dependent kinase and caspase-1 activation precedes mitochondrial dysfunction in fumarylacetoacetate-induced apoptosis. FASEB J..

[20] Jorquera R., Tanguay R.M. (2001). Fumarylacetoacetate, the metabolite accumulating in hereditary tyrosinemia, activates the erk pathway and induces mitotic abnormalities and genomic instability. Hum. Mol. Genet..

[21] Tanguay R.M., Jorquera R., Poudrier J., St-Louis M. (1996). Tyrosine and its catabolites: From disease to cancer. Acta Biochim. Pol..

[22] Endo F., Kubo S., Awata H., Kiwaki K., Katoh H., Kanegae Y., Saito I., Miyazaki J., Yamamoto T., Jakobs C. (1997). Complete rescue of lethal albino c14cos mice by null mutation of 4-hydroxyphenylpyruvate dioxygenase and induction of apoptosis of hepatocytes in these mice by *in vivo* retrieval of the tyrosine catabolic pathway. J. Biol. Chem..

[23] Kubo S., Sun M., Miyahara M., Umeyama K., Urakami K., Yamamoto T., Jakobs C., Matsuda I., Endo F. (1998). Hepatocyte injury in tyrosinemia type 1 is induced by fumarylacetoacetate and is inhibited by caspase inhibitors. Proc. Natl. Acad. Sci. USA.

[24] Luijerink M.C., Jacobs S.M., van Beurden E.A., Koornneef L.P., Klomp L.W., Berger R., van den Berg I.E. (2003). Extensive changes in liver gene expression induced by hereditary tyrosinemia type I are not normalized by treatment with 2-(2-nitro-4-trifluoromethylbenzoyl)-1,3-cyclohexanedione (ntbc). J. Hepatol..

[25] Thimm E., Richter-Werkle R., Kamp G., Molke B., Herebian D., Klee D., Mayatepek E., Spiekerkoetter U. (2012). Neurocognitive outcome in patients with hypertyrosinemia type I after long-term treatment with ntbc. J. Inherit. Metab. Dis..

[26] Schiff M., Broue P., Chabrol B., de Laet C., Habes D., Mention K., Sarles J., Spraul A., Valayannopoulos V., Ogier de Baulny H. (2012). Heterogeneity of follow-up procedures in french and belgian patients with treated hereditary tyrosinemia type 1: Results of a questionnaire and proposed guidelines. J. Inherit. Metab. Dis..

[27] Al-Dhalimy M., Overturf K., Finegold M., Grompe M. (2002). Long-term therapy with ntbc and tyrosine-restricted diet in a murine model of hereditary tyrosinemia type I. Mol. Genet. Metab..

[28] Vogel A., van Den Berg I.E., Al-Dhalimy M., Groopman J., Ou C.N., Ryabinina O., Iordanov M.S., Finegold M., Grompe M. (2004). Chronic liver disease in murine hereditary tyrosinemia type 1 induces resistance to cell death. Hepatology.

[29] Bergeron A., Jorquera R., Orejuela D., Tanguay R.M. (2006). Involvement of endoplasmic reticulum stress in hereditary tyrosinemia type I. J. Biol. Chem..

[30] Orejuela D., Jorquera R., Bergeron A., Finegold M.J., Tanguay R.M. (2008). Hepatic stress in hereditary tyrosinemia type 1 (ht1) activates the akt survival pathway in the *fah*^−/−^ knockout mice model. J. Hepatol..

[31] Viktorsson K., Lewensohn R., Zhivotovsky B. (2005). Apoptotic pathways and therapy resistance in human malignancies. Adv. Cancer Res..

[32] Grompe M., Lindstedt S., al-Dhalimy M., Kennaway N.G., Papaconstantinou J., Torres-Ramos C.A., Ou C.N., Finegold M. (1995). Pharmacological correction of neonatal lethal hepatic dysfunction in a murine model of hereditary tyrosinaemia type I. Nat. Genet..

[33] Rohde M., Daugaard M., Jensen M.H., Helin K., Nylandsted J., Jaattela M. (2005). Members of the heat-shock protein 70 family promote cancer cell growth by distinct mechanisms. Genes Dev..

[34] Scieglinska D., Piglowski W., Mazurek A., Malusecka E., Zebracka J., Filipczak P., Krawczyk Z. (2008). The hspa2 protein localizes in nucleoli and centrosomes of heat shocked cancer cells. J. Cell Biochem..

[35] Garg M., Kanojia D., Seth A., Kumar R., Gupta A., Surolia A., Suri A. (2010). Heat-shock protein 70–2 (hsp70-2) expression in bladder urothelial carcinoma is associated with tumour progression and promotes migration and invasion. Eur. J. Cancer.

[36] Tahara T., Arisawa T., Shibata T., Yamashita H., Nakamura M., Yoshioka D., Okubo M., Maruyama N., Kamano T., Kamiya Y. (2012). Role of heat-shock protein (hsp) 70–2 genotype in peptic ulcer in japanese population. Hepatogastroenterology.

[37] Kolb S.J., Snyder P.J., Poi E.J., Renard E.A., Bartlett A., Gu S., Sutton S., Arnold W.D., Freimer M.L., Lawson V.H. (2010). Mutant small heat shock protein b3 causes motor neuropathy: Utility of a candidate gene approach. Neurology.

[38] Hosoda A., Kimata Y., Tsuru A., Kohno K. (2003). Jpdi, a novel endoplasmic reticulum-resident protein containing both a bip-interacting j-domain and thioredoxin-like motifs. J. Biol. Chem..

[39] Cunnea P.M., Miranda-Vizuete A., Bertoli G., Simmen T., Damdimopoulos A.E., Hermann S., Leinonen S., Huikko M.P., Gustafsson J.A., Sitia R. (2003). Erdj5, an endoplasmic reticulum (er)-resident protein containing dnaj and thioredoxin domains, is expressed in secretory cells or following er stres. J. Biol. Chem..

[40] Charette S.J., Lavoie J.N., Lambert H., Landry J. (2000). Inhibition of daxx-mediated apoptosis by heat shock protein 27. Mol. Cell Biol..

[41] Sherman M. (2010). Major heat shock protein hsp72 controls oncogene-induced senescence. Ann. NY Acad. Sci..

[42] Matsushima-Nishiwaki R., Takai S., Adachi S., Minamitani C., Yasuda E., Noda T., Kato K., Toyoda H., Kaneoka Y., Yamaguchi A. (2008). Phosphorylated heat shock protein 27 represses growth of hepatocellular carcinoma via inhibition of extracellular signal-regulated kinase. J. Biol. Chem..

[43] Yasuda E., Kumada T., Takai S., Ishisaki A., Noda T., Matsushima-Nishiwaki R., Yoshimi N., Kato K., Toyoda H., Kaneoka Y. (2005). Attenuated phosphorylation of heat shock protein 27 correlates with tumor progression in patients with hepatocellular carcinoma. Biochem. Biophys. Res. Commun..

[44] Bruey J.M., Ducasse C., Bonniaud P., Ravagnan L., Susin S.A., Diaz-Latoud C., Gurbuxani S., Arrigo A.P., Kroemer G., Solary E. (2000). Hsp27 negatively regulates cell death by interacting with cytochrome c. Nat. Cell Biol..

[45] Bruey J.M., Paul C., Fromentin A., Hilpert S., Arrigo A.P., Solary E., Garrido C. (2000). Differential regulation of hsp27 oligomerization in tumor cells grown *in vitro* and *in vivo*. Oncogene.

[46] Guay J., Lambert H., Gingras-Breton G., Lavoie J.N., Huot J., Landry J. (1997). Regulation of actin filament dynamics by p38 map kinase-mediated phosphorylation of heat shock protein 27. J. Cell Sci..

[47] Oya-Ito T., Liu B.F., Nagaraj R.H. (2006). Effect of methylglyoxal modification and phosphorylation on the chaperone and anti-apoptotic properties of heat shock protein 27. J. Cell Biochem..

[48] Guo K., Gan L., Zhang S., Cui F.J., Cun W., Li Y., Kang N.X., Gao M.D., Liu K.Y. (2012). Translocation of hsp27 into liver cancer cell nucleus may be associated with phosphorylation and o-glcnac glycosylation. Oncol. Rep..

[49] Brunet M., Didelot C., Subramaniam S., Rérole A.L., de Thonel A., Garrido C., Calderwood S., Sherman M., Ciocca D. (2007). Hsp70 and hsp27 as pharmacological targets in apoptosis modulation for cancer therapy. Heat Shock Proteins in Cancer.

[50] Bryantsev A.L., Chechenova M.B., Shelden E.A. (2007). Recruitment of phosphorylated small heat shock protein hsp27 to nuclear speckles without stress. Exp. Cell Res..

[51] Zhang D., Wong L.L., Koay E.S. (2007). Phosphorylation of ser78 of hsp27 correlated with her-2/neu status and lymph node positivity in breast cancer. Mol. Cancer.

[52] Gottlieb E., Vander Heiden M.G., Thompson C.B. (2000). Bcl-x(l) prevents the initial decrease in mitochondrial membrane potential and subsequent reactive oxygen species production during tumor necrosis factor alpha-induced apoptosis. Mol. Cell Biol..

[53] Gottlieb R.A. (2000). Mitochondria: Execution central. FEBS Lett..

[54] Koehler B.C., Scherr A.L., Lorenz S., Urbanik T., Kautz N., Elssner C., Welte S., Bermejo J.L., Jager D., Schulze-Bergkamen H. (2013). Beyond cell death—Antiapoptotic bcl-2 proteins regulate migration and invasion of colorectal cancer cells *in vitro*. PLoS One.

[55] Michaud W.A., Nichols A.C., Mroz E.A., Faquin W.C., Clark J.R., Begum S., Westra W.H., Wada H., Busse P.M., Ellisen L.W. (2009). Bcl-2 blocks cisplatin-induced apoptosis and predicts poor outcome following chemoradiation treatment in advanced oropharyngeal squamous cell carcinoma. Clin. Cancer Res..

[56] Sartorius U.A., Krammer P.H. (2002). Upregulation of Bcl-2 is involved in the mediation of chemotherapy resistance in human small cell lung cancer cell lines. Int. J. Cancer.

[57] Tabuchi Y., Matsuoka J., Gunduz M., Imada T., Ono R., Ito M., Motoki T., Yamatsuji T., Shirakawa Y., Takaoka M. (2009). Resistance to paclitaxel therapy is related with Bcl-2 expression through an estrogen receptor mediated pathway in breast cancer. Int. J. Oncol..

[58] Savry A., Carre M., Berges R., Rovini A., Pobel I., Chacon C., Braguer D., Bourgarel-Rey V. (2013). Bcl-2-enhanced efficacy of microtubule-targeting chemotherapy through bim overexpression: Implications for cancer treatment. Neoplasia.

[59] Rousseau S., Houle F., Kotanides H., Witte L., Waltenberger J., Landry J., Huot J. (2000). Vascular endothelial growth factor (vegf)-driven actin-based motility is mediated by vegfr2 and requires concerted activation of stress-activated protein kinase 2 (sapk2/p38) and geldanamycin-sensitive phosphorylation of focal adhesion kinase. J. Biol. Chem..

[60] Kindas-Mugge I., Trautinger F. (1994). Increased expression of the m(r) 27,000 heat shock protein (hsp27) in *in vitro* differentiated normal human keratinocytes. Cell Growth Differ..

[61] Lanneau D., Brunet M., Frisan E., Solary E., Fontenay M., Garrido C. (2008). Heat shock proteins: Essential proteins for apoptosis regulation. J. Cell Mol. Med..

[62] Garrido C., Brunet M., Didelot C., Zermati Y., Schmitt E., Kroemer G. (2006). Heat shock proteins 27 and 70: Anti-apoptotic proteins with tumorigenic properties. Cell Cycle.

[63] Beere H.M. (2004). “The stress of dying”: The role of heat shock proteins in the regulation of apoptosis. J. Cell Sci..

[64] Nylandsted J., Gyrd-Hansen M., Danielewicz A., Fehrenbacher N., Lademann U., Hoyer-Hansen M., Weber E., Multhoff G., Rohde M., Jaattela M. (2004). Heat shock protein 70 promotes cell survival by inhibiting lysosomal membrane permeabilization. J. Exp. Med..

[65] Lee J.S., Lee J.J., Seo J.S. (2005). Hsp70 deficiency results in activation of c-jun n-terminal kinase, extracellular signal-regulated kinase, and caspase-3 in hyperosmolarity-induced apoptosis. J. Biol. Chem..

[66] Gao T., Newton A.C. (2002). The turn motif is a phosphorylation switch that regulates the binding of hsp70 to protein kinase c. J. Biol. Chem..

[67] Arrigo A.P. (2005). Heat shock proteins as molecular chaperones. Med. Sci. (Paris).

[68] Hwang T.S., Han H.S., Choi H.K., Lee Y.J., Kim Y.J., Han M.Y., Park Y.M. (2003). Differential, stage-dependent expression of hsp70, hsp110 and bcl-2 in colorectal cancer. J. Gastroenterol. Hepatol..

[69] Milicevic Z.T., Petkovic M.Z., Drndarevic N.C., Pavlovic M.D., Todorovic V.N. (2007). Expression of heat shock protein 70 (hsp70) in patients with colorectal adenocarcinoma—Immunohistochemistry and western blot analysis. Neoplasma.

[70] Wang X.P., Qiu F.R., Liu G.Z., Chen R.F. (2005). Correlation between clinicopathology and expression of heat shock protein 70 and glucose-regulated protein 94 in human colonic adenocarcinoma. World J. Gastroenterol..

[71] Romani A.A., Crafa P., Desenzani S., Graiani G., Lagrasta C., Sianesi M., Soliani P., Borghetti A.F. (2007). The expression of hsp27 is associated with poor clinical outcome in intrahepatic cholangiocarcinoma. BMC Cancer.

[72] Glaessgen A., Jonmarker S., Lindberg A., Nilsson B., Lewensohn R., Ekman P., Valdman A., Egevad L. (2008). Heat shock proteins 27, 60 and 70 as prognostic markers of prostate cancer. APMIS.

[73] Yu H.J., Chang Y.H., Pan C.C. (2013). Prognostic significance of heat shock proteins in urothelial carcinoma of the urinary bladder. Histopathology.

[74] Evans C.G., Chang L., Gestwicki J.E. (2010). Heat shock protein 70 (hsp70) as an emerging drug target. J. Med. Chem..

[75] Massey A.J., Williamson D.S., Browne H., Murray J.B., Dokurno P., Shaw T., Macias A.T., Daniels Z., Geoffroy S., Dopson M. (2010). A novel, small molecule inhibitor of hsc70/hsp70 potentiates hsp90 inhibitor induced apoptosis in hct116 colon carcinoma cells. Cancer Chemother. Pharmacol..

[76] Strasser A., Harris A.W., Bath M.L., Cory S. (1990). Novel primitive lymphoid tumours induced in transgenic mice by cooperation between myc and bcl-2. Nature.

[77] Strasser A., Harris A.W., Cory S. (1993). E mu-bcl-2 transgene facilitates spontaneous transformation of early pre-b and immunoglobulin-secreting cells but not t cells. Oncogene.

[78] McDonnell T.J., Troncoso P., Brisbay S.M., Logothetis C., Chung L.W., Hsieh J.T., Tu S.M., Campbell M.L. (1992). Expression of the protooncogene bcl-2 in the prostate and its association with emergence of androgen-independent prostate cancer. Cancer Res..

[79] Charlotte F., L’Hermine A., Martin N., Geleyn Y., Nollet M., Gaulard P., Zafrani E.S. (1994). Immunohistochemical detection of bcl-2 protein in normal and pathological human liver. Am. J. Pathol..

[80] Papadimitriou C.S., Costopoulos J.S., Christoforidou B.P., Kotsianti A.J., Karkavelas G.S., Hytiroglou P.M., Koufogiannis D.J., Nenopoulou H.E. (1997). Expression of bcl-2 protein in human primary breast carcinomas and its correlation with multifocality, histopathological types and prognosis. Eur. J. Cancer.

[81] Heiser D., Labi V., Erlacher M., Villunger A. (2004). The bcl-2 protein family and its role in the development of neoplastic disease. Exp. Gerontol..

[82] Jeon B.S., Yoon B.I. (2012). Altered expression of cellular bcl-2 in the progression of hamster cholangiocarcinogenesis. Sci. World J..

[83] Hohfeld J., Jentsch S. (1997). Grpe-like regulation of the hsc70 chaperone by the anti-apoptotic protein bag-1. EMBO J..

[84] Takayama S., Xie Z., Reed J.C. (1999). An evolutionarily conserved family of hsp70/hsc70 molecular chaperone regulators. J. Biol. Chem..

[85] Sondermann H., Scheufler C., Schneider C., Hohfeld J., Hartl F.U., Moarefi I. (2001). Structure of a bag/hsc70 complex: Convergent functional evolution of hsp70 nucleotide exchange factors. Science.

[86] Sreedhar A.S., Csermely P. (2004). Heat shock proteins in the regulation of apoptosis: New strategies in tumor therapy: A comprehensive review. Pharmacol. Ther..

[87] Luders J., Demand J., Hohfeld J. (2000). The ubiquitin-related bag-1 provides a link between the molecular chaperones hsc70/hsp70 and the proteasome. J. Biol. Chem..

[88] Alberti S., Demand J., Esser C., Emmerich N., Schild H., Hohfeld J. (2002). Ubiquitylation of bag-1 suggests a novel regulatory mechanism during the sorting of chaperone substrates to the proteasome. J. Biol. Chem..

[89] Demand J., Alberti S., Patterson C., Hohfeld J. (2001). Cooperation of a ubiquitin domain protein and an e3 ubiquitin ligase during chaperone/proteasome coupling. Curr. Biol..

[90] Tanguay R.M., Wu Y., Khandjian E.W. (1993). Tissue-specific expression of heat shock proteins of the mouse in the absence of stress. Dev. Genet..

