# Supplementary material for: Heat Shock Response Associated with Hepatocarcinogenesis in a Murine Model of Hereditary Tyrosinemia Type I
Source: Cancers (Basel). 2014 Apr 23;6(2):998–1019. doi: 10.3390/cancers6020998 (PMC4074813; doi:10.3390/cancers6020998)
Supplement: Supplementary File 1 — Supplementary Data (PDF, 133 KB) [file cancers-06-00998-s001.pdf]

Cancers **2014**, *6*, 1-x manuscript; doi:10.3390/cancers60x000x

OPEN ACCESS

cancers

ISSN 2072-6694
www.mdpi.com/journal/cancers

Supplementary Data

Heat Shock Response Associated with Hepatocarcinogenesis in a Murine Model of Hereditary Tyrosinemia Type I

Francesca Angileri, Geneviève Morrow, Vincent Roy, Diana Orejuela † and Robert M. Tanguay *

Laboratory of Cell and Developmental Genetics, Department of Molecular Biology, Medical Biochemistry and Pathology, Institut de Biologie Intégrative et des Systèmes (IBIS) and PROTEO, 1030 avenue de la médecine, Université Laval, Québec G1V 0A6, Canada; E-Mails: francesca.angileri.1@ulaval.ca (F.A.); genevieve.morrow@fmed.ulaval.ca (G.M.); vincent.roy.15@ulaval.ca (V.R.); diana.orejuela@efort.org (D.O.)

† Current address: EFORT Head Office, ZA La Pièce 2, Rolle 1180, Switzerland.

* Author to whom correspondence should be addressed; E-Mail: robert.tanguay@ibis.ulaval.ca; Tel.: +1-418-656-3339; Fax: +1-418-656-7176.

Received: 6 December 2013; in revised form: 15 March 2014 / Accepted: 3 April 2014 / Published:

Table S1. Summary of the molecules deregulated according to the process involved.

Top Diseases and Functions	Score	Molecules in Network
Amino Acid Metabolism, Small Molecule Biochemistry, Molecular Transport	30	ACSL4, ALDH18A1, BUB1, CAMK2A, Ccl6, CDR2, CPOX, DHX9, DNAJB9, DNASE1, EIF2AK3, GFPT1, GLS, GRAP2, HIST1H1E, IPO7, LAMB, LARS, MCM6, ME2, MTHFR, MYC, PRODH, RYR2, SCEL, SLC11A1, SLC16A1, SLC1A4, SLC1A5, SLC3A2, SYCP3, TBPL1, TIAM1, TNFRSF10A, ZIC2
Cell Signaling, Cancer, Neurological Disease	25	ALCAM, AMPH, APBB1IP, ARHGEF6, ATPIF1, CTNNA1, DMBT1, DOK3, EDARADD, ETV5, FABP6, FXYD5, GSR, GTPBP4, GZMK, HIP1, IER3, KRAS, MLKL, NCAM2, NFkB(complex), NUP98, P2RX3, PKMYT1, PLCE1, RAB31, RASSF2, RRAS2, SENP6, SLC2A5, SRC (family), TJP2, TNFAIP8, TNIP1, UHMK1
Cancer, Cellular Movement, Tumor Morphology	25	ACSS1, Akr1c19, ASPN, BCAM, CALB2, CHST8, COL4A6, Collagen typeI, EMP2, EMX2, FBLN5, FLRT2, FLT1, FZD2, GAL3ST1, GDF6, GFAP, GPR12, GPRC5B, HBEGF, ITGA6, ITGB1, ITGB4, KDELR3, LAMA5, Laminin, LOXL1, MFAP4, NRXN1, ONECUT2, OPN3, P2RY14, PDGFC, SLC16A9, TGFB1

Table S1. *Cont.*

Top Diseases and Functions	Score	Molecules in Network
Lipid Metabolism, Small Molecule Biochemistry, Carbohydrate Metabolism	25	ABCB1, ABCD2, AFP, AQP5, ASAH1, BTG3, CDC6, Cox8b, CRABP2, CYP3A5, DNTT, EFCAB7, ELOVL7, ERMAP, G6PD, GABPB2, Hist2h4, Histoneh3, Histoneh4, HOXB1, HSPA4L, Irg1, MCM3, MEIS1, NR0B1, NRIP1, NSD1, PNLIPRP2, PYGL, Rhox5, RPS6KA3, SPTLC2, SUV39H2, THRSP, WNT4
Humoral Immune Response, Protein Synthesis, Infectious Disease	24	15LOX, AHNAK, BST1, CAPG, CD83, Cd24a, CHST3, CLEC10A, CRIP1, CYSLTR1, GLIPR2, GPR34, HCK, HLA-DMA, ICOSLG, Iga, IgG2b, IGJ, IL4, IL36B, IL36G, IMPDH1, IRF2BP2, LTB, MAOA, MAP3K14, PDCD1, PFKP, PKIB, SERPINB6, SPON2, Sprr1a, THOP1, TNFRSF9, ULBP1
Cardiovascular System Development and Function, Connective Tissue Disorders, Organismal Injury and Abnormalities	24	ACTR3, CCL13, COL1A1, COL1A2, COL3A1, COL4A2, COL8A1, CRHR1, CRHR2, CTGF, DDR1, DSP, Dynamin, FAS, Fibrinogen, FLI1, GLIS2, GRIP1, ITGAV, ITGB6, KIF5B, LTBP2, LTBP3, MGP, MMP12, MMP13, MMP14, NCK2, NT5E, PCSK5, PCSK6, PDGF-CC, PTGIR, SERPINH1, UCN3
Cell Cycle, Cardiovascular Disease, Organismal Injury and Abnormalities	24	ADAM8, Aldose Reductase, ALOX5, ANLN, APOB, APOBEC1, CD84, CDCA3, CDCA5, CDCA8, CHTF18, CSF2, ERCC6L, FBXO5, FIGNL1, KIF11, KNTC1, LDL, LDLcholesterol, LIPC, MSR1, NEK2, NUSAP1, OSMR, PHGDH, PON1, RACGAP1, Reg3g, SGOL1, Slfn2, SPAG5, TPX2, TRG, UBD, UBE2C
Embryonic Development, Tissue Morphology, Cancer	24	Ap2alpha, BAG2, CA3, CCND1, CDK5, RAP2, CLCN5, CyclinE, CYP1A1, CYP1B1, DAB2, DDX52, DVL3, EPN2, EPS15, GCN1L1, Gm4794/Sult3a1, Hdac, KLF6, LRP2, MCAM, MKI67, MT1E, PITX2, QKI, RIF1, ROCK2, SHH, SLC9A3, SOCS2, TBX1, TBX3, TCF21, TUBB6, TYMS, VIL1
Cellular Movement, Nervous System Development and Function, Cardiovascular System Development and Function	24	14-3-3, ABLIM1, CHD9, CXCR4, CYP11B1, DBP, FLNA, FOXC1, IFI44, LPIN2, Mcpt8, MGST2, MKL1, MOGAT2, MYH9, NEDD4L, NFAT (complex), NFE2L3, Ngp, NR2F2, NRP1, PA2G4, PGLYRP1, PRG2, SCNN1A, STK17B, TCR, TMEM27, TNFRSF12A, Tpm4, VAMP2, YWHAE, YWHAH, YWHAZ
Hereditary Disorder, Inflammatory Disease, Free Radical Scavenging	22	5430435G22Rik, ABCC1, Calcineurin protein(s), CD2AP, CNN2, CSNK2A1, CTSK, CYBA, CYBB, CYP2E1, GRB10, IGFBP6, JAM2, JAM3, LAPTM5, LAX1, LMX1B, MAFB, MAP3K6, MYH2, NADPHoxidase, NCF1, NCF2, NCF4, NEDD4, NUAK1, P38MAPK, PFN1, PIK3CG, PLA2G7, PLA2G4A, RAC2, SH3KBP1, TICAM2, Tropomyosin
Cell Cycle, DNA Replication, Recombination, and Repair, Cellular Growth and Proliferation	22	AKR1B1, BUB3, CCNE2, CCNI, Cdc2, CDCA2, CDKN1A, CDKN1C, CHAF1A, CHEK1, CLSPN, CNOT6L, CTBP2, CyclinB, DNAJB4, DTL, EDNRA, EXO1, HERC2, MBNL2, MCM2, NGFR, PCM1, PCNA, PP2A, PRDM16, PSAP, RAD18, RB1, RHOC, RRM2, RRM2B, thymidine kinase, TP53INP1, UHRF1

Table S1. *Cont.*

Top Diseases and Functions	Score	Molecules in Network
Cell Cycle, Cellular Assembly and Organization, DNA Replication, Recombination, and Repair	22	AURKB, BRCA1, BUB1B, CCNB1, CCNB2, CENPA, CENPE, CENPF, CKAP2, CMKLR1, CollagentypeIV, CYR61, DDIT4, ENTPD1, FBN1, Fibrin, FOXM1, GAS6, GTSE1, IL-2R, LAMA4, LMO4, LOX, LOXL2, MMP9, PLK4, POSTN, PRC1, Rb, RBBP8, ROBO1, SFTPD, STXBP4, TNFRSF18, TP63
Cellular Assembly and Organization, Cellular Function and Maintenance, Tissue Development	22	ABI1, CFL1, EPS8, ERK, F Actin, GCNT, GSN, HIF3A, KCNB1,KCNV2, LIMK1, MCF2L, Mlc, MVP, NF1, PAK1, PDGFBB, PDGFB, PDGFRB, PLAT, PTPRC, PTPRE, PTPRS, RGS5, RHOJ, RND3, ROCK1, SLC20A1, SPRED1, SPRED2, SSH1, TAOK1, TBC1D4, TGM2, THY1
Cellular Development, Cellular Growth and Proliferation, Hair and Skin Development and Function	22	ADAM17, ADAMTS5, Alpha catenin, AREG/AREGB, BCL11B, BGN, BICC1, BMPER, BTC, Cadherin, Calmodulin, CDH11, CHKA, CSNK1E, DCN, EGFR, EGR2, ID4, IQGAP1, LAMC1, MMP7, MMP8, NEO1, NFATC2, NID1, NID2, NIPA2, OSR1, OSTF1, PAFAH1B1, RHOB, Smad, TGFBR1, TGFBR2, TTF1
Cellular Movement, Cell-To-Cell Signaling and Interaction, Hematological System Development and Function	22	ACSL1, Caspase 3/7, CCL4, CD44, CD63, COL5A1, COL5A2, CXADR, CYP4A11, EHHADH, EMILIN1, FBLIM1, Filamin, HLA-DMB, HLA-DR, ICAM1, IRAK3, ITGA4, ITGAM, ITGAX, ITGB2, KREMEN1, LGALS3, LTBP1, LTF, LUM, LY6D, MSN, MYADM, PDK4, PTP4A3, SAA, TEAD2, VCAM1, WWTR1
Cellular Function and Maintenance, Hematological System Development and Function, Inflammatory Response	22	26s Proteasome, ACTG1, Actin, ASB9, ASNS, BRCA2, CA4, CD47, CFTR, CUL5, DAB1, ENC1, EPB42, HDAC2, HSD3B1, Hsp70, IL7, IL12 (family), IL7R, Pfn2, PPP2R2B, RAB25, RBBP7, RORA, S100A8, S100A9, S100A11, Scd2, SLA, SLC5A4, SMARCC1, SMARCE1, TOP2A, USP9Y, VIM
Humoral Immune Response, Protein Synthesis, Developmental Disorder	20	ADAM15, BCR (complex), BLNK, CD22, CDH2, CLDN1, DAPP1, DGKH, DHCR24, EFNB1, FANCA, FRK, FUT8, GALNT3, GPC3, GSTA1, HSF2, HSPA1A/HSPA1B, HSPB1, IgD, Igg3, IL18R1, Jnk, MAP4K5, Mbl1, MGAT3, PAWR, PLS1, Pmca, PROM1, RNASEL, SAV1, SOX4, SRD5A1, TNFRSF21
Organ Morphology, Humoral Immune Response, Protein Synthesis	20	ANXA3, ARHGAP22, CABIN1, CACNA2D1, CACNB3, CALU, CBFB, CD48, CD53, Collagentype III, G-protein beta, Gp49a/Lilrb4, GPR160, GPR171, IER5, Ige, IGF2, IGF1R, IVNS1ABP, KCNK5, KIF20A, KIF5C, LAD1, MAP3K8, NFKB2, PRKCDBP, RELB, RNASE3, RNMT, SNCG, SNN, TGFBI, Troponint, UBE2V2, Ubiquitin
Cell Death and Survival, Cardiac Necrosis/Cell Death, Cell Morphology	20	1600029D21Rik, A2M, ANKRD1, BCL2A1, calpain, creatine kinase, CTPS1, ELMO1, FSTL1, GAS7, GATA4, GSK3B, HSD3B2, LCN2, LMNA, LMNB1, Mitochondrial complex 1, NKX2-5, NPPA, NR2C2, PAPPA, Pka, PRRX1, PRUNE, PTPN7, Rac, RCN2, REST, SGCA, SLIT3, SOX9, SPARC, SYN1, WISP2, ZFR
Molecular Transport, Small Molecule Biochemistry, Drug Metabolism	20	ABCC2, Alp, ALT, ARHGAP18, BMP6, BMP2K, Creb, CYP2A13, CYP2C8, CYP39A1, CYP7B1, CYP8B1, DIO1, EP300, FRMD4B, GSTM1, Ikb, MATN2, MMP1, MXD3, NR1I3, OGN, RUNX2, SERPINE1, SESN3, SHOC2, SLC39A10, Slco1a4, Smad2/3, SOX5, SPAG6, Sult1a1, SYNPO, TMEM119, TMPRSS2

Table S1. *Cont.*

Top Diseases and Functions	Score	Molecules in Network
Gene Expression, Cell Morphology, Organismal Survival	19	BAZ1A, C1QA, C3AR1 CDK6, CDKN2B, CITED2, COL2A1, Collagen Alpha1, COMMD3BMI1, CXCL14, Cyclin A, CYGB, DACH1, E2f, EHF, estrogen receptor, FABP4, Gsk3, HMGA2, HMGB2, JUN, MAPK7, MEF2C, MEF2D, MYOZ1, N-cor, N4BP1, NLRP12, Pmaip1, PRKAR2B, RB1CC1, SKIL, SMURF2, SNAI1, TWIST1
Cellular Movement, Hematological System Development and Function, Immune Cell Trafficking	19	ADCY, ADRB2, ATF3, CCL2, CCL7, Ccl8, CCL11, CCL17, CCL3L1/CCL3L3, CCR2, Chi3l3/Chi3l4, Collagen(s), CREB5, Ctbp, EGR1, GRIA1, HGF, IFN alpha/beta, KCNJ3, MAP2K1/2, Mcpt1, MSC, NCR1, NDRG1, NFKBIZ, Nrg1, P2RY12, Pdgf Ab, PLAUR, PRSS8, ST14, TIMP1, TIMP2, TLR7, TREM3
Cellular Movement, Immune Cell Trafficking, Cell-To-Cell Signaling and Interaction	19	ABCA3, CD14, CDH1, CSF1, CXCL6, EDN1, FGFBP1, GATA6, GRN, HLA-B, Hsp27, IL22, IL22RA1, KITLG, LMCD1, LRAT, Mapk, NFIA, PTPN11, PTPRO, RAP2B, RBP1, RBP7, Retnla, S100A4, SFTPC, SLIT2, STAT5a/b,Tgf beta, THBD, Tlr, TP53I11, UGP2, VCL
Lipid Metabolism, Molecular Transport, Small Molecule Biochemistry	19	ACACA, ACACB, ANGPT2, ARPC1B, AUTS2, CASP1, CNTN1, CPT1, DKK2, HMG CoAsynthase, HMGCR, HMGCS1, IFI27, IL1, Interferon alpha, MHC-Class I (complex), NDRG2, p70S6k, PANX1, PRKAA1, S100A6, S100G, SATB2, SERPINB9, SLC19A1, SLC8A1, SOAT1, SPARCL1, SPP1, SREBF2, TLR1, TMSB10/TMSB4X, TRPV5, USP6NL, VDR
Cell-To-Cell Signaling and Interaction, Cellular Movement, Hematological System Development and Function	17	ABCC5, CCL20, CD97, CD209, Cdk, CLEC6A, CLEC7A, EGR3, ELF3, EPHX2, F2RL1, Fcgr2, Gm-csf, GPNMB, IL6, IL23, IL-1R, IL17F, IL17RC, IL1R2, IL1RAP, LGMN, LSP1, MMP28, NEU3, OLR1, PBK, PDLIM2, Ras homolog, TAC1, TACR1, TIMP3, Tnf receptor, TRAFD1, UNC5B

© 2014 by the authors; licensee MDPI, Basel, Switzerland. This article is an open access article distributed under the terms and conditions of the Creative Commons Attribution license (http://creativecommons.org/licenses/by/3.0/).
